# Living well: Empirically developed structural equation model for healthy and effective self-regulation

**DOI:** 10.1016/j.ijchp.2023.100375

**Published:** 2023-03-05

**Authors:** Benedict Heblich, Orestis Terzidis, Marcos González M, Katherina Kuschel, Mouzzam Mukadam, Marius Birkenbach

**Affiliations:** a Karlsruhe Institute of Technology, Karlsruhe, Germany; b Universidad Austral de Chile, Puerto Montt, Chile; c CENTRUM Católica Graduate Business School, Lima, Peru; d Pontificia Universidad Católica del Perú, Lima, Peru

**Keywords:** Self-determination theory, Psychological needs satisfaction, Psychological well-being, Subjective well-being, Mindfulness, Personal values

## Abstract

The purpose of this study is to develop and empirically test a structural equation model (SEM) for healthy and effective self-regulation based on the propositions of self-determination theory (SDT). A cross-sectional data sample (*N* = 6,705) is used to test the model. The results of the SEM demonstrate good to excellent global fit indices (RMSEA = 0.06, SRMR = 0.04 CFI = 0.97, TLI/NNFI = 0.95) and excellent local fit indices (*p* < 0.001). It is acknowledged that longitudinal and experimental research designs will be necessary to infer causal effects. However, based on the strong theoretical and empirical grounding of the model, indications for causal effects are discussed beyond correlational relations. The local fit indices imply that autonomy of goals, intrinsic values orientation, mindfulness, and the newly integrated construct clarity about personal values positively affect psychological needs satisfaction and facets of subjective and psychological well-being. Additionally, they indicate that mindfulness and clarity about personal values have the greatest benefits on individual health, well-being, and effectiveness. These results are crucial as they emphasize the significant role of mindfulness in healthy and effective self-regulation. Furthermore, they put the spotlight on a rather new construct; clarity about personal values. By having transferred the knowledge base of SDT into an empirically derived model of healthy and effective self-regulation, this study provides well-grounded indications of how health, well-being, and effectiveness in individuals may be fostered. These indications offer new insights for theory building and practical interventions in domains like psychotherapy, healthcare, organizations, sports, and education.

## Introduction

In the scope of self-determination theory (SDT), there is a body of research that investigates healthy and effective self-regulation in an integrated way (Deci and Ryan, 2000; Ryan, Huta and Deci, 2008; Schultz and Ryan, 2015). In this body, four constructs are intensely researched: autonomous motivation (e.g., Sheldon, 2014, Sheldon and Elliot, 1999), intrinsic life-goals orientation (e.g. Grouzet et al., 2005 on intrinsic aspirations; Kasser, 2004; Sortheix and Schwartz, 2017 on intrinsic personal values), mindfulness (e.g., Brown and Ryan, 2003), and psychological needs satisfaction (e.g., Ryan and Deci, 2017). Ryan et al. (2008) and Schultz and Ryan (2015) state that the three self-regulatory key ingredients autonomous motivation (the “why”), intrinsic life-goals orientation (the “what”), and mindfulness (the “how”) lead to basic psychological needs satisfaction as an outcome. According to Ryan et al. (2008), the proposed concept of healthy and effective self-regulation could be seen as grounded in a rather Aristotelean view on happiness (eudaimonia).

Furthermore, Ryan et al. (2008) make empirically grounded propositions that other constructs besides psychological needs satisfaction are also outcome variables of healthy self-regulation in the scope of SDT. They can be subdivided into variables that describe positive effects on the individual and the societal level. On the individual level, they describe positive effects like higher subjective and psychological well-being (Ryan et al., 2008). They explicitly emphasize positive affect and satisfaction with life (Diener et al., 2009) as two possible outcomes that could be subsumed under subjective well-being. Furthermore, they emphasize meaning in life (Steger, Frazier, Oishi and Kaler, 2006) and subjective vitality (Ryan and Frederick, 1997) that could be subsumed under psychological well-being (Ryan et al., 2008). Besides, they describe faster goal progress on the individual level (Sheldon and Elliot, 1999). On the societal level, they describe positive effects such as prosocial and ecological-friendly behavior (Ryan et al., 2008).

Ryan et al. (2008) provide an excellent overview of constructs and causations that represent healthy and effective self-regulation. However, this overview is based on a body of empirical studies that investigate only single or small fragments of healthy and effective self-regulation. Ryan et al. (2008) put together the fragments into a theoretical model of healthy and effective self-regulation. The current study aims to take the first step toward the empirical validation of the overall model. It integrates the proposed constructs and causations into a comprehensive SEM and tests it with a huge cross-sectional data set (*N* = 6705). We highlight that it is not possible to validate causal relations with cross-sectional data. However, the overall SEM and the integrated causal relations are well-grounded in existing research, and a large cross-sectional sample can be considered a strong test for falsification. If the model and the causal relations are *not* falsified based on local and global fit indices, this supports indications for causalities (Kline, 2015; Wunsch, Russo and Mouchart, 2010). The current study initiates SEM by developing the conceptual model and research hypotheses based on theoretical and empirical studies mainly made in the scope of SDT. Therefore, the constructs and causal relations theorized by Ryan et al. (2008) are integrated. Furthermore, the model is refined with constructs, operationalizations, and causations from recent research studies.

## Conceptual model and research hypotheses

**Fig. 1:**
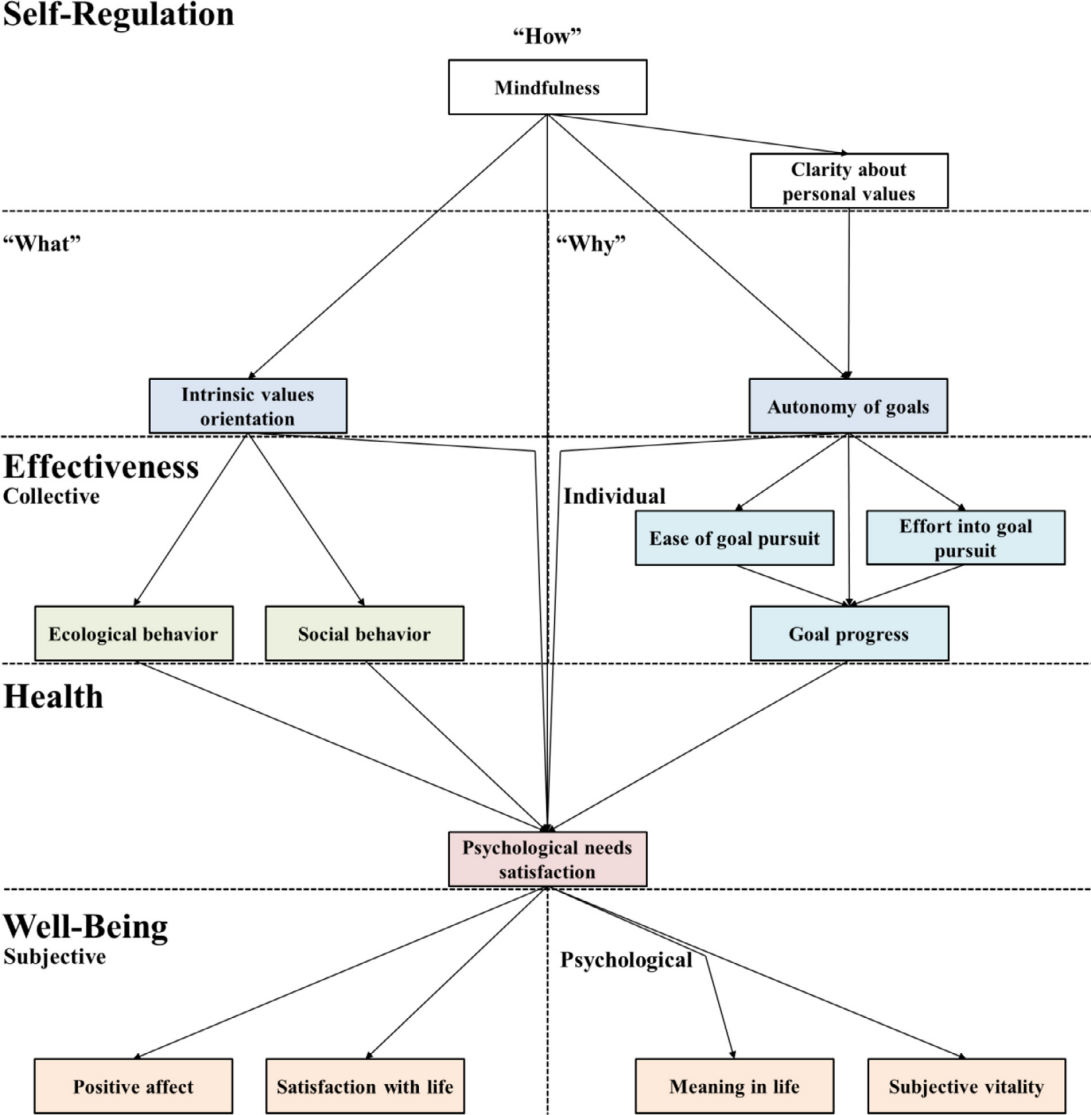
Transfer of SDT's understanding of healthy and effective self-regulation into a hypothesized structural equation model.

Fig. 1 gives an overview of the SEM of healthy and effective self-regulation that we empirically developed based on existing literature. Fig. 1 shows all proposed constructs and causations. The empirically derived hypotheses building is described step-by-step in the following.

### The output: transferring SDT's understanding of health, well-being, and effectiveness into the structural equation model

In SDT, the satisfaction of the three basic psychological needs (autonomy, relatedness, and competence) is seen as the essential nutrient for the healthy functioning of the human (Deci and Ryan, 2000). This perspective is supported by an extensive body of empirical studies showing positive relations of psychological needs satisfaction with constructs that refer to health, well-being, and engagement (see Deci and Ryan, 2000). Based on Ryan et al. (2008), the current study conceptualizes health in the scope of SDT as the satisfaction of the three basic psychological needs and conceptualizes well-being as the causal consequence on the two essential well-being dimensions of hedonic and eudaimonic well-being (see Fig. 1). A body of empirical studies indicates that psychological needs satisfaction leads to hedonic well-being, e.g., subjective well-being (Neubauer and Voss, 2016; Ryan et al., 2008; Sheldon, Ryan and Reis, 1996) as well as to eudaimonic well-being, e.g., psychological well-being (Ryan et al., 2008; Sheldon et al., 1996). Therefore, positive affect (Diener et al., 2009) and satisfaction with life (Diener, Emmons, Larsen and Griffin, 1985) are integrated as two dimensions of subjective well-being. Furthermore, meaning in life (Steger et al., 2006) and subjective vitality (Ryan and Frederick, 1997) are integrated as two dimensions of psychological well-being (see Fig. 1).

Motivated by Ryan et al.’s (2008) conceptualization of healthy and effective self-regulation, the current study integrates also concepts that refer to individual and collective effectiveness as output variables. Individual effectiveness is integrated to measure effects that refer to more individualized measures of individual progress in life. For the concrete conceptualization, this study was inspired by Emmons (1986) and Sheldon and Elliot (1999), therefore referring to the goal progress of individuals (see Fig. 1). Collective effectiveness is integrated to measure the effects of self-regulatory processes on the planet and the people. Promoting ecologically and socially sustainable development seems essential to face global challenges like social inequality and climate change (United Nations, 2015). To conceptualize collective effectiveness, this study is inspired by Ryan et al. (2008) and refers to the daily ecological and daily social behavior of an individual (based on the EBQ by Butenko and Schwartz, 2013) (see Fig. 1).

Sheldon and Elliot (1999) indicate through structural equation modeling (SEM) with longitudinal data that goal progress leads to psychological needs satisfaction. However, a study by Sheldon and Kasser (1998) implies that the amount of increased well-being depends on the degree of “organismic congruence” (p. 1319), which recent studies would describe as the autonomy of goals. Nevertheless, the results indicate a causal relationship between goal progress and psychological needs satisfaction, independent of the goal's autonomy. Thus, the present study hypothesizes that goal progress causes psychological needs satisfaction (see Fig. 1). For ecological behavior, prior results by Brown and Kasser (2005) and Kasser (2009) indicate that ecological behavior leads to higher levels of psychological needs satisfaction and higher levels of well-being. Given the described central role of psychological needs satisfaction on well-being by Ryan et al. (2008), this study argues that the positive effects of ecological behavior on well-being could be mediated through psychological needs satisfaction. Thus, it hypothesizes that ecological behavior causes psychological needs satisfaction (see Fig. 1). For social behavior, a study by Steger et al. (2008) indicates that social behavior leads to higher levels of well-being. Based on Deci and Ryan (2000) and Ryan et al. (2008), this study argues that the positive effects of social behavior on well-being could be mediated through psychological needs satisfaction. Therefore, it is hypothesized that social behavior causes psychological needs satisfaction. Based on these studies, the effectiveness constructs are positioned as preceding health and well-being (see Fig. 1), although they are also seen as output variables of healthy and effective self-regulation.

### The input: transferring the “why”, “what”, and “how” of healthy and effective self-regulation into the structural equation model

#### The “Why”

In the scope of SDT, it is stated that the degree of self-determination could be used to specify a “why” of self-regulation that leads to health, well-being, and effectiveness (Ryan et al., 2008; Schultz and Ryan, 2015). Self-determined actions are stronger led by autonomous goals than by controlled goals. In specific, autonomous goals are rather motivated by authentic interests and personal values, while controlled goals are rather motivated by external rewards and punishments or introjected feelings such as fear or shame (Sheldon, 2014; Sheldon and Elliot, 1999). Sheldon and Elliot (1999) show that individuals with more autonomous goals make faster progress and have higher psychological needs satisfaction. The goal progress seems mediated by sustained effort to pursue an autonomous goal (Sheldon and Elliot, 1999; Smith, Ntoumanis and Duda, 2007; Smith et al., 2011). However, Werner, Milyavskaya, Foxen-Craft and Koestner (2016) found that the mediational effect does not necessarily have to be the effort invested into autonomous goals but could be the ease and naturalness of goal pursuit. Based on these studies, we conceptualize the “why” of healthy and effective self-regulation through the autonomy of goals which should lead to effectiveness and psychological needs satisfaction (see Fig. 1). In addition, ease of goal pursuit (Werner et al., 2016) and effort into goal pursuit (Sheldon and Elliot, 1999) are integrated as mediators between autonomy of goals and goal progress (see Fig. 1).

#### The “What”

**Fig. 2:**
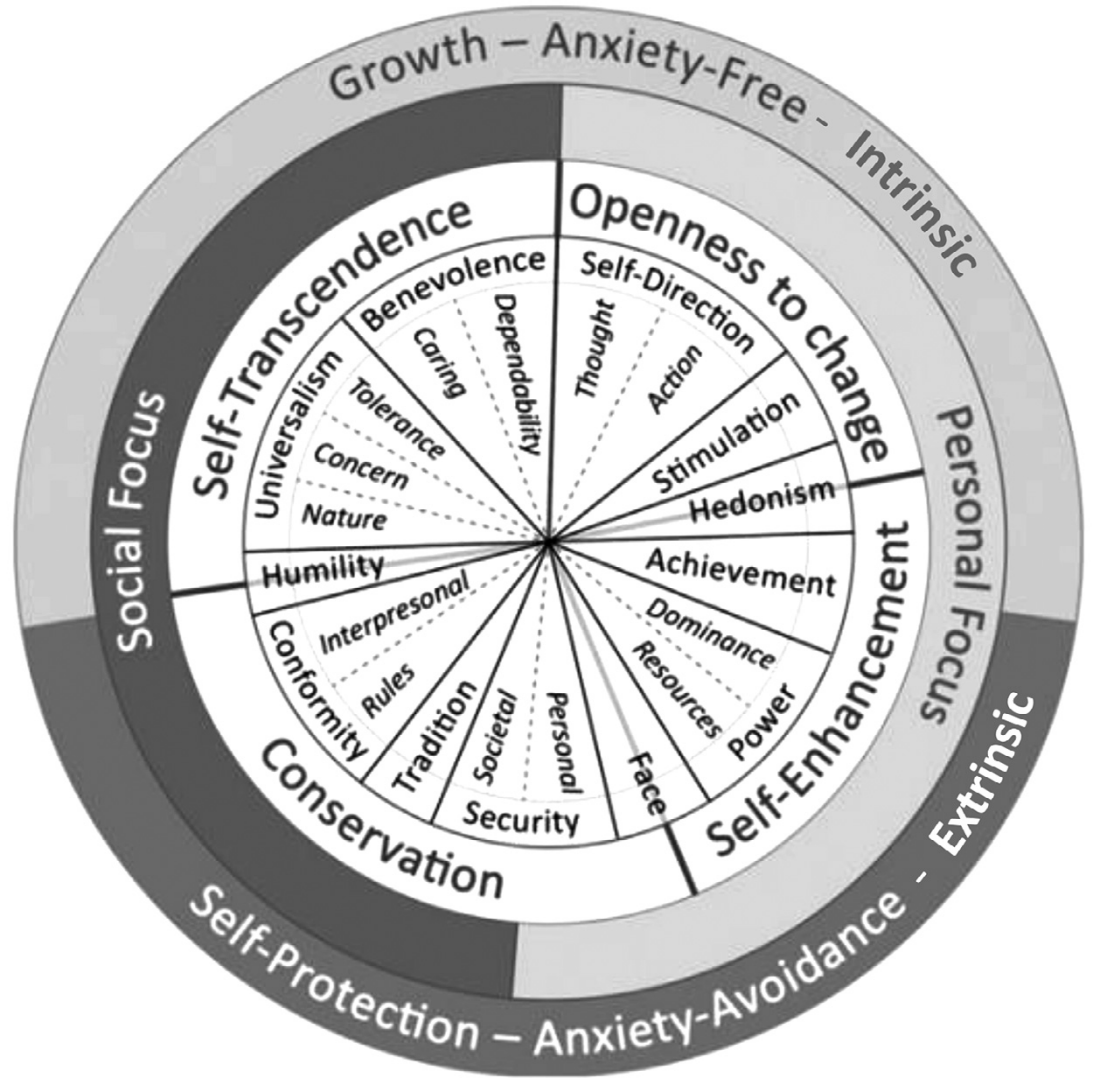
Refined universal continuum of human values by Schwartz (visualization based on Cieciuch et al., 2014; Sagiv and Schwartz, 2022; Schwartz, 1992, 2016; Schwartz et al., 2012).

In the scope of SDT, it is stated that the content of an individual's life goals, often referred to as aspirations or personal values, could be used to specify the “what” of self-regulation that leads to health, well-being, and effectiveness. A fundamental paper in this area was published by Grouzet et al. (2005). It empirically divides life goals into intrinsic and extrinsic aspirations. Intrinsic aspirations are conceptualized to arise from the innate natural human tendency to achieve effectiveness, connectedness, and coherence (Deci and Ryan, 2000). By that, they are characterized to be those kinds of life goals that rather lead to satisfying basic psychological needs (Deci and Ryan, 2000, Kasser, 2004). Examples of intrinsic aspirations are affiliation, self-acceptance, community, and physical health (Grouzet et al., 2005). Extrinsic aspirations are conceptualized to arise from the wish to get external signs of worth or contingent approval. By that, they are said to be less likely to lead to the satisfaction of basic psychological needs (Deci and Ryan, 2000). Examples of extrinsic aspirations are conformity, popularity, image, and financial success (Grouzet et al., 2005). Empirical studies in the scope of SDT show that the pursuit of intrinsic life goals is positively related to the satisfaction of the basic psychological needs and other concepts of well-being and health (e.g., Kasser and Ryan, 1993, 1996, 2001; Kiaei and Reio, 2014). There is also a body of conceptual and empirical studies (Heblich, 2021; Heblich and Terzidis, 2016; Kasser, 2002; Sagiv and Schwartz, 2022; Sortheix and Schwartz, 2017) that transfers the concept of intrinsic and extrinsic life goals to the universal continuum of human values (see Fig. 2). Although the results are often weakly significant and not consistent across cultures, it is indicated for most cultures that the upper, growth-oriented values (from universalism to hedonism), could be interpreted as rather intrinsic and the lower, self-protecting values (from conformity to power), as rather extrinsic. Humility and achievement may have both characteristics (Sagiv and Schwartz, 2022; Schwartz, 2016). Furthermore, studies in the scope of the theory of planned behavior (Ajzen, 1991; Sheeran, Norman and Orbell, 1999) indicate that valued intentions (also called attitudinal intention) are associated with referring behavior. As we conceptualized collective effectiveness as social (McHoskey, 1999) and ecologically friendly (Brown and Kasser, 2005; Sheldon and McGregor, 2000) behavior, we see them as two intrinsic behavior facets motivated by the referring universalistic values of concern and nature. Based on the conceptualization of intrinsic values and the studies that have been made on the positive effects of intrinsic life goals on dimensions of well-being, health, and behavior; this study positions intrinsic values orientation as preceding and causing psychological needs satisfaction, whereas social and ecological behavior are seen as mediators on the behavioral level (see Fig. 1).

#### The “How”

The third input concept of healthy and effective self-regulation in the scope of SDT addresses “how” an individual can acquire autonomous motivation (the “Why”) and intrinsic life goals (the “What”) to achieve health, well-being, and effectiveness. Empirical studies in the scope of SDT (e.g., Brown and Ryan, 2003) indicate that mindfulness fosters autonomous goals and intrinsic life goals, and psychological needs satisfaction (Ryan et al., 2008; Schultz and Ryan, 2015). In SDT, mindfulness is conceptualized as “a receptive state of mind wherein attention, informed by a sensitive awareness of what is occurring at the moment, plainly observes internal (e.g., psychological and somatic experiences) and external events that are taking place” (Brown and Ryan, 2003; Kabat-Zinn, 2003 cited by Schultz and Ryan, 2015, p. 84). Besides, it is often described as pre-reflexive and non-evaluative awareness (Ryan et al., 2008; Schultz and Ryan, 2015). Based on the conceptualization of mindfulness and the studies that have been made on the positive effects of mindfulness, this study emphasizes mindfulness as being essential for healthy and effective self-regulation. It is characterized as a construct that directly or indirectly influences all discussed constructs (see Fig. 1). Furthermore, we integrate a related concept that is rather new to SDT research. Schultz and Ryan (2015) describe that there is a reflexive state of mind that can follow the pre-reflexive state of mindfulness. Based on Brown and Ryan (2003), they argue that mindfulness is associated with self-knowledge and self-insight (e.g. Silvia and Duval, 2001). We see self-knowledge as a consequence of the pre-reflexive state of mind, mindfulness, and as a mediator between mindfulness and autonomy of goals (see Fig. 1). In the model, we integrate a specific type of self-knowledge: clarity about personal values (Trompetter, 2014). By integrating this type of self-knowledge, the model acknowledges the body of research that has been made in the scope of Acceptance and Commitment Therapy (ACT, Hayes, 2004, 2016), which emphasizes the importance and positive effects of recognition and knowledge of personal values. Having clarity about personal values enables to define and commit to self-integrated, autonomous goals, and empowers to endure the pursuit until achievement (Hayes, 2004; Hayes et al., 2016; Trompetter, 2014).

## Method

### Procedure and participants

As a research design for developing and testing an SEM, we followed the eight recommended steps by Weiber and Mühlhaus (2014). These are building hypotheses and the model, the conceptualization of constructs, operationalization of constructs, quality test of model-constructs, model estimation with SPSS AMOS, evaluation of the model, interpretation of results, and modification of the model structure based on modification indices. The cross-sectional data for empirical SEM-testing was gathered through an online questionnaire that integrates fifteen well-tested operationalizations for each of the constructs, which resulted in an overall number of 156 items for the constructs (see Table 2). Besides, 4 demographic questions were included (see Table 4). Participants completed the questionnaire self-selected as part of the personality assessment “Core Values Finder” (Heblich, Mukadam and Birkenbach, 2022), which is a research-based personality assessment that provides the participants an overview of their personal values tendencies based on the Portraits Values Questionnaire Revised (PVQ-RR, Schwartz and Butenko, 2014). A total of 12,221 participants completed the questionnaire over a period of 4 years. All data was anonymized and gathered in compliance with the ethical principles of the researching institution. To ensure data quality, the sample was purified. As recommended for online questionnaires, four control questions were used to exclude participants for which strong satisficing effects were obvious (Krosnick, 1991). Furthermore, only fully completed questionnaires with all items answered were taken into regard, leading to a final sample of *N* = 6705 individuals. The questionnaire was usable in English and German. 5056 (75.4%) participants answered the English questionnaire and 1649 (24.6%) the German version. Table 1 provides the descriptive characteristics of the sample. Summarizing the descriptive characteristics, the sample consisted of substantially more women (66.4%) than men; a big proportion of participants is in the range of 16 to 40 years of age (76.4%); most participants live in English-speaking countries like the USA (14.3%), Australia (6.2%), United Kingdom (5.5%), or in Germany (24.0%), whereas the proportion that is subsumed under “Other” is widely spread among 155 other countries. Concerning occupation, the biggest proportion of participants is employed for wages (45.0%), followed by being students (34.0%), or being self-employed (14.8%).

**Table 1:** Sample Description.

Participant characteristic	N	Percentage
Gender		
Female	4452	66.4
Male	2220	33.1
Non-Binary	33	0.5
Range of age		
11–15	327	4.9
16–20	1072	16.0
21–25	1213	18.1
26–30	1178	17.6
31–35	951	14.2
36–40	703	10.5
41–45	485	7.2
46–50	347	5.2
> 50	429	6.4
Current place of living		
Germany	1610	24.0
USA	962	14.3
Australia	417	6.2
United Kingdom	366	5.5
India	351	5.2
Other	2998	44.7
Occupation		
Employed for wages	2733	45.0
Student	2068	34.0
Self-employed	897	14.8
Unemployed	415	6.8
Pupil	209	3.4
Housemaker	135	2.2
Apprentice	80	1.3
Pensioner	32	0.5
Other	136	2.2

### Measures

As the current study integrates 15 scales (156 items) for the constructs, it presents their operationalization with referring characteristics in the form of a table (see Table 2). For scales that are not common operationalizations for the referring constructs and are outside of the scope of SDT, explanations below the table are provided.

**Table 2:** Construct and referring scales with characteristics.

Construct	Scale	Author(s) Year	Items	Answer Scale	Score
Autonomy of goals	Relative Autonomy Index (RAI)	Sheldon and Elliot (1999)	4 for each goal	Likert scale from 1 (strongly disagree) to 6 (strongly agree)	Relation of autonomous reasons to controlled reasons
Clarity about personal values	Valued Living Scale (VLS)	Trompetter (2014)	4	Likert scale from 1 (strongly disagree) to 6 (strongly agree)	Mean
Ease of goal pursuit	Ease of Goal Pursuit	Werner et al. (2016)	1 for each goal	Likert scale from 1 (strongly disagree) to 6 (strongly agree)	Mean
Ecological behavior	Everyday Behavior Questionnaire (EBQ) – Universalism Nature	Butenko and Schwartz (2013)	4	Likert scale from 0 (never) to 4 (always)	Mean
Effort into goal pursuit	Effort into Goal Pursuit	Sheldon and Elliot (1999)	1 for each goal	Likert scale from 1 (strongly disagree) to 6 (strongly agree)	Mean
Goal description	Personal Strivings (PS)	Emmons (1986)	1 for each goal	Qualitative	–
Goal progress	Goal Progress	Sheldon and Elliot (1999)	1 for each goal	Likert scale from 1 (strongly disagree) to 6 (strongly agree)	Mean
Intrinsic values orientation	Revised Portraits Values Questionnaire (PVQ-RR)	Schwartz and Butenko (2014)	57	Likert scale from 1 (not like me at all) to 6 (very much like me)	Relative intrinsic life-goals importance
Meaning in life	Perceived Meaning in Life Scale (PMLS)	Steger et al. (2006)	5	Likert scale from 1 (strongly disagree) to 7 (strongly agree)	Mean
Mindfulness	Mindfulness Attention Awareness Scale (MAAS)	Brown and Ryan (2003)	15	Likert scale from 1 (almost always) to 6 (almost never)	Mean
Positive affect	Scale for Positive And Negative Experience (SPANE)	Diener et al. (2009)	12	Likert scale from 1 (Never or very rarely) to 5 (very often or always)	Relative frequency of positive experiences
Psychological needs satisfaction	Psychological Well-being Scale (MIDUS – II; Autonomy, Environmental Mastery, and Positive relations with others)	Ryff (1989); Ryff and Keyes (1995)	21	Likert scale from 1 (strongly disagree) to 6 (strongly agree)	Mean
Satisfaction with life	Satisfaction with Life Scale (SWLS)	Diener et al. (1985); Kobau, Sniezek, Zack, Lucas and Burns (2010)	5	Likert scale from 1 (strongly disagree) to 7 (strongly agree)	Sum
Social behavior	Everyday Behavior Questionnaire (EBQ) – Universalism Concern	Butenko and Schwartz (2013)	4	Likert scale from 0 (never) to 4 (always)	Mean
Subjective vitality	Subjective Vitality Scale (SVS)	Ryan and Frederick (1997)	5	Likert scale from 1 (strongly disagree) to 7 (strongly agree)	Mean

#### Operationalizations from outside of SDT

For *clarity about personal values*, there was no adequate operationalization in the scope of SDT. Therefore, a well-developed scale from ACT (Hayes et al., 2016) was integrated: the Valued Living Scale (VLS, Trompetter, 2014). The included items (e.g., “I have values that give my life more meaning”) measure the recognition and knowledge of personal values. Referring to Trompetter (2014), the four items with the highest factor loadings were included to measure the concept that we labeled as clarity about personal values.

*Intrinsic values orientation* is operationalized based on the Revised Portraits Values Questionnaire (PVQ-RR, Schwartz and Butenko, 2014). This operationalization was used to offer participants a more comprehensive range of possible trans-situational than is available in the aspiration index (Grouzet et al., 2005; Kasser and Ryan, 2001). Although there are no consistent results throughout cultures regarding the relation of the importance of specific personal values with well-being, there is a tendency in most cultures (based on Bilsky and Schwartz, 1994; Sagiv and Schwartz, 2022). It is indicated that nine growth-oriented values (from universalism-nature to hedonism) could be interpreted as rather intrinsic, and eight self-protecting values could be interpreted as rather extrinsic (from Conformity-interpersonal to Power-dominance). The values of humility and achievement could be interpreted as relatively neutral (see Fig. 2). To measure intrinsic values orientation, the same calculation approach as in the aspiration index was used (Grouzet et al., 2005; Kasser and Ryan, 2001), which is calculating the mean over all intrinsic values.

To measure *social* respectively *ecological behavior*, the two dimensions “Universalism-Concern” and “Universalism-Nature” of the *Everyday Behavior Questionnaire (EBQ,* Butenko and Schwartz, 2013*)* are used. It measures the behavioral dimension of referring personal values as two dimensions of intrinsic behavior.

To measure the *satisfaction of the three basic psychological needs*, three dimensions of the Psychological Well-being Scale (MIDUS-II, Ryff, 1989M Ryff and Keyes, 1995) were used. This study did not use the Basic Needs Satisfaction in General – Scale (BNSG-S, Deci and Ryan, 2000). This scale could be seen as the first choice to measure the three basic psychological needs as it was developed by the founders of SDT, Richard Ryan and Edward Deci, and is widely used. However, in the current study, it is argued that the used items conceptualize the need for autonomy mainly in the sense of independence (e.g., item 4: ‘There were people telling me what I had to do.’) (Sheldon and Hilpert, 2012). Ryan and Deci (2013) expressively emphasize that the need for autonomy is not similar to independence. In line with other well-cited studies like Brown and Ryan (2003), this study uses the Psychological Well-being Scale (MIDUS-II, Ryff, 1989; Ryff and Keyes, 1995). It better fits for autonomy in particular because it encompasses not only items that could be subsumed under independence but also items that measure what Ryan and Deci (2013) call wholeheartedness behind the behavior, regardless of whether one is independent in the situation (e.g., item 7: ‘I judge myself by what I think is important, not by the values of what others think is important.’) (Ryff, 1989; Ryff and Keyes, 1995). Therefore, and in line with other well-cited studies like Brown and Ryan (2003), the need for autonomy is measured through the dimension autonomy, the need for competence is measured through the dimension of environmental mastery, and the need for relatedness is measured through the dimension of positive relations to others. Thus, the part of the scale that this study applies encompasses 21 items (7 items for each need).

## Results

**Fig. 3:**
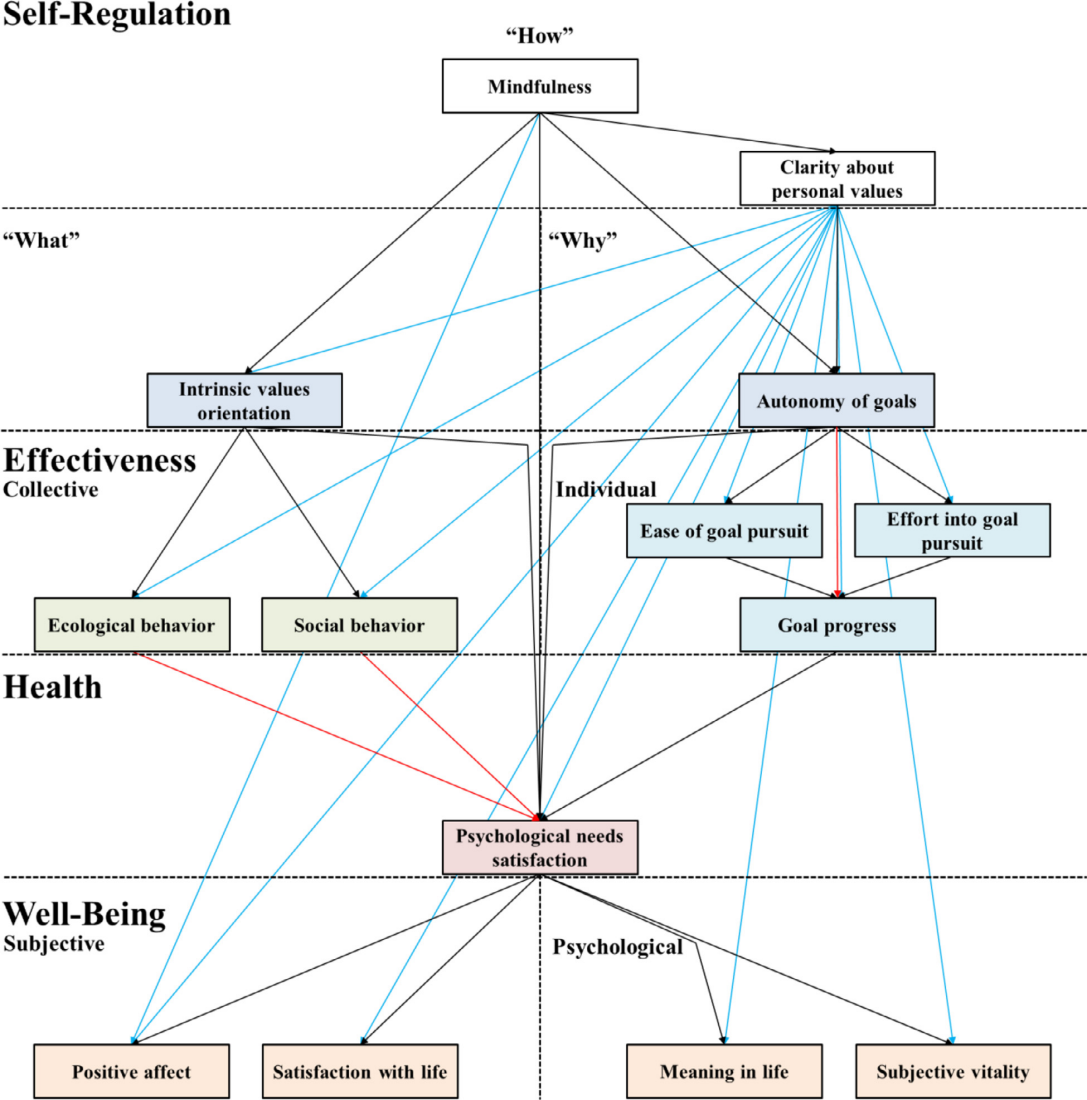
Hypothesized SEM with the paths that were rejected (red) and paths that were added (blue).

**Fig. 4:**
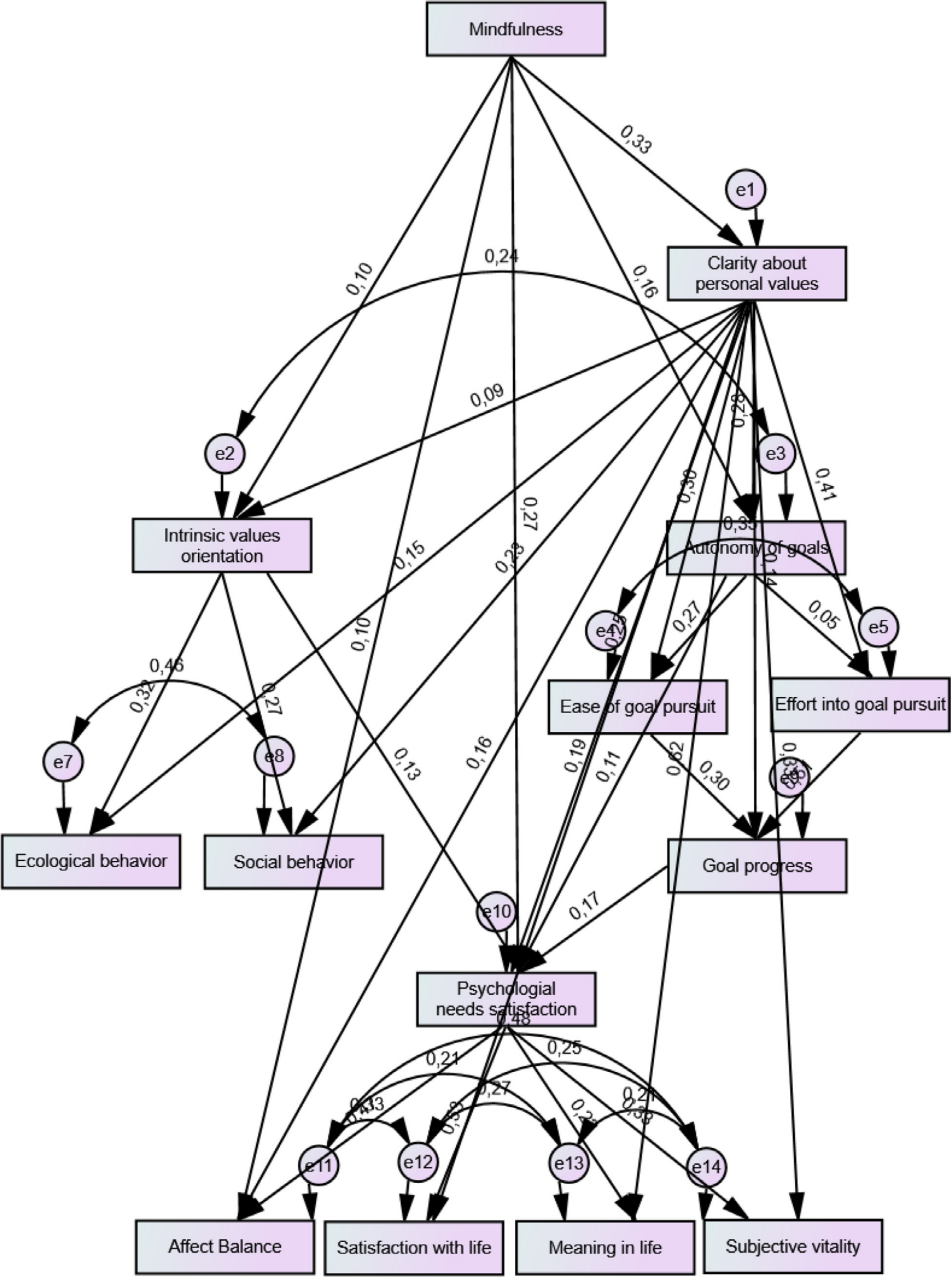
Structural equation model in SPSS AMOS with standardized estimates.

The Maximum Likelihood Estimator (MLE) is applied using SPSS AMOS to estimate the SEM. This estimation method assumes univariate and multivariate normality (Weiber and Mühlhaus, 2014). Looking at the Kolmogorov-Smirnoff and Shapiro Wilk test results (recommended *N* ≤ 2000) as well as at the critical ratios of the multivariate kurtosis, it is indicated that the constructs are not normally distributed. However, normality tests are seen as too sensitive for large sample sizes (*N* > 2000) (Royston, 1982). Referring to the central limit theorem for large sample sizes (Kwak and Kim, 2017) as well as by analyzing the histograms and qq-plots, we conclude a normal distribution for the variables. Homburg and Klarmann (2006) and Weiber and Mühlhaus (2014) recommend adapting the estimated SEMs based on modification indices to improve the global model fit to a reasonable point. Therefore, in this study, adaptions were limited until an excellent global model fit was reached for most indices. As global fit indices, Homburg and Klarmann (2006) suggest using RMSEA (Root Mean Square Residual), CFI (Comparative Fit Index), and NNFI (Nonnormed Fit Index), which is also called TLI (Tucker Lewis Index). Homburg and Klarmann (2006) argue, based on Browne and Cudeck (1992) as well as on Schermelleh-Engel, Moosbrugger and Müller (2003), that an RMSEA < 0.05 could be interpreted as an excellent model fit, while an RMSEA < 0.1 could be described as an acceptable model fit. CFI and NNFI should be higher than 0.9. Furthermore, when items with different Likert-scale ranges are used, it is recommended to also test for the standardized root mean square residual (SRMR), for which an excellent model fit is a value less than 0.05 (Hooper, Coughlan and Mullen, 2008). Table 3 shows the results for these global fit indices. Fig. 3 shows the paths that had to be rejected (red) and added (blue) in this process. Fig. 4 shows the resulting SEM in SPSS AMOS with the referring standardized direct effects in Table 5 and the standardized total effects in.

**Table 3:** Global fit indices for the final structural equation model.

**RMSEA**	**SRMR**	**CFI**	**NNFI/TLI**
.06	.04	.97	.95

The four global fit indices meet their specific cut-off level requirement (CFI and NNFI/TLI > 0.9, RMSEA < 0.1, SRMR < 0.05). Furthermore, all local fit indices have a high level of significance (*p* < 0.001). Most paths that were added based on the modification indices stem from the construct clarity about personal values. The highest direct effects of self-regulatory constructs on well-being, health, or effectiveness constructs stem from mindfulness and clarity about personal values.

Furthermore, Pearson correlation coefficients are provided in Table 4.

**Table 4:** Pearson correlation coefficients and referring p labels, ***: *p* < 0.001.

The four global fit indices meet their specific cut-off level requirement (CFI and NNFI/TLI > 0.9, RMSEA < 0.1, SRMR < 0.05). Furthermore, all local fit indices have a high level of significance (*p* < 0.001). Most paths that were added based on the modification indices stem from the construct clarity about personal values. The highest direct effects of self-regulatory constructs on well-being, health, or effectiveness constructs stem from mindfulness and clarity about personal values.

## Discussion

Overall, the SEM shows a *good to excellent global model fit* and an *excellent local model fit* (*p* < 0.001 for all direct effects, see Table 5). In combination with the large sample size, the diversity of sample characteristics, and the subsequent theoretical and empirical rooting of the model, the good to excellent statistical results allow for deriving outstandingly well-validated and deep grounded scientific interpretations.

**Table 5:** Standardized direct effects and referring p labels, ***: *p* < 0.001.

**Paths**			**Estimate**	**P label**
Mindfulness	→	Clarity about personal values	.33	***
	→	Autonomy of goals	.16	***
	→	Intrinsic values orientation	.10	***
	→	Psychological needs satisfaction	.27	***
	→	Affect balance	.10	***
Clarity about personal values	→	Autonomy of goals	.29	***
	→	Intrinsic values orientation	.09	***
	→	Ease of goal pursuit	.30	***
	→	Effort into goal pursuit	.41	***
	→	Goal progress	.17	***
	→	Ecological behavior	.15	***
	→	Social behavior	.23	***
	→	Psychological needs satisfaction	.25	***
	→	Affect balance	.16	***
	→	Satisfaction with life	.19	***
	→	Meaning in life	.62	***
	→	Subjective Vitality	.33	***
Intrinsic values orientation	→	Ecological behavior	.32	***
	→	Social behavior	.27	***
	→	Psychological needs satisfaction	.13	***
Autonomy of goals	→	Ease of goal pursuit	.27	***
	→	Effort into goal pursuit	.05	***
	→	Psychological needs satisfaction	.11	***
Ease of goal pursuit	→	Goal progress	.30	***
Effort into goal pursuit	→	Goal progress	.50	***
Goal progress	→	Psychological needs satisfaction	.11	***
Psychological needs satisfaction	→	Positive affect	.47	***
	→	Satisfaction with life	.53	***
	→	Meaning in life	.22	***
	→	Subjective vitality	.33	***

### The output: health, well-being, and effectiveness

Ryan et al. (2008) propose that health could be conceptualized at the core by satisfying the three basic psychological needs (autonomy, competence, and relatedness). The nutriment of these essentials should lead to subjective and psychological well-being. These propositions are supported in the SEM through the strong and highly significant direct effects from psychological needs satisfaction to two facets of subjective well-being: Satisfaction with life and Positive affect as well as to two facets of psychological well-being: Presence of meaning in life and Subjective vitality (see Table 5). Motivated by Ryan et al.’s (2008) conceptualization of healthy and effective self-regulation, the current study also integrated concepts that refer to individual and collective effectiveness as output variables, preceding the health and well-being variables. Individual effectiveness, measured through goal progress was supported in having significant effects on all health and well-being constructs (see Table 6).

The four global fit indices meet their specific cut-off level requirement (CFI and NNFI/TLI > 0.9, RMSEA < 0.1, SRMR < 0.05). Furthermore, all local fit indices have a high level of significance (*p* < 0.001). Most paths that were added based on the modification indices stem from the construct clarity about personal values. The highest direct effects of self-regulatory constructs on well-being, health, or effectiveness constructs stem from mindfulness and clarity about personal values.

However, those effects are weak. This supports the empirical studies that indicate that not mainly progress but autonomy (Sheldon, 1999, 2014) and the content of a goal influence health, well-being, and effectiveness positively (Kasser and Ryan, 1993, 1996, 2001; Kasser, 2016). In contrast to the propositions of Ryan et al. (2008), ecological and social behavior as operationalizations of collective effectiveness did not have a significantly positive effect on the health and well-being constructs. The relations were therefore deleted in the adaption process of the SEM. These results open questions like how healthy it is to not only pursue but act on self-transcendent, universalistic values. The results of the current study indicate that acting on these specific values has no effect on health and well-being. Furthermore, the operationalization that was used (EBQ, Butenko and Schwartz, 2013) could be discussed as many questions focus on how frequently a person is talking about the referring topic (e.g., “Discuss suffering and poverty in the world with another person.”, Butenko and Schwartz, 2013). Therefore, further studies that may test other operationalizations for ecologically friendly and social behavior are necessary to analyze the relation to health and well-being constructs. Overall, the mainly excellent global fit indices show that the model seems to grasp the output dimensions validly. Future studies can build on the integrated and state-of-the-art conceptualization and operationalization of health, well-being, and effectiveness.

### The input: the “Why”, “What”, and “How”

Ryan et al.’s (2008) core propositions are that mindfulness (the “how”), autonomy of goals (the “why”), and intrinsic values orientation (the “what”) foster health, well-being, and effectiveness. This core of SDT's propositions is supported by the SEM's direct and total effects (Table 5).

The four global fit indices meet their specific cut-off level requirement (CFI and NNFI/TLI > 0.9, RMSEA < 0.1, SRMR < 0.05). Furthermore, all local fit indices have a high level of significance (*p* < 0.001). Most paths that were added based on the modification indices stem from the construct clarity about personal values. The highest direct effects of self-regulatory constructs on well-being, health, or effectiveness constructs stem from mindfulness and clarity about personal values.

As mediational constructs between autonomy of goals and goal progress, “effort” into (Sheldon, 1999) as well as “ease” of the goal pursuit (Werner et al., 2016) were supported. What stands out is that from the three input variables, mindfulness has by a clear margin the strongest effects on health, well-being, and effectiveness. For the autonomy of goals and intrinsic life-goals orientation, the effects on the output are only weak in comparison. This is a crucial result as it points out that the role of mindfulness, although widely researched in other domains, is essential in comparison to the other self-regulatory processes derived from SDT.

Furthermore, the study indicates that one psychological construct that has not been emphasized in the scope of SDT has a significantly positive effect on health, well-being, and effectiveness. Namely, the newly integrated construct “*clarity about personal values*” has strong direct and total effects on constructs operationalized under health, well-being, and effectiveness (Table 5 and Table 6).

**Table 6:** Standardized Total Effect and referring p labels; **: *p* < 0.01.

The four global fit indices meet their specific cut-off level requirement (CFI and NNFI/TLI > 0.9, RMSEA < 0.1, SRMR < 0.05). Furthermore, all local fit indices have a high level of significance (*p* < 0.001). Most paths that were added based on the modification indices stem from the construct clarity about personal values. The highest direct effects of self-regulatory constructs on well-being, health, or effectiveness constructs stem from mindfulness and clarity about personal values.

In the adaption process of the SEM the modification indices pointed to integrate a relation to all subsequent constructs (Fig. 3 and Fig. 4). From our perspective, this is the most relevant insight of this study as it is a rather new finding in the research domain.

Having clarity about personal values, in other words, having clarity about one's integrated core of motivation seems crucial for health, well-being, and effectiveness of individuals. Overall, the drafted SEM implies that individuals, who strive for healthy and effective self-regulation, can benefit from exercising mindfulness and finding clarity about their personal values. Based on the findings on mindfulness in combination with clarity about personal values, we interpret that mindfulness can help individuals to bring unconscious layers of personality into consciousness and to integrate them into their life. In specific terms, the non-judgmental and observing character of mindfulness could help get clarity about and integrate personal values into one's life.

Besides, mindfulness helps discover intrinsic core values as well as set autonomous goals that are in congruence with them. The total effects indicate that this process, directly and indirectly, fosters health, well-being, and effectiveness. Although the findings concerning clarity about personal values are rather new, they are in alignment with and support new therapy approaches like ACT (Hayes et al., 2016) that focus on helping patients to be mindful and to create and execute values-based commitments. Beyond the positive implications of the SEM for individual health, well-being, and effectiveness, it also indicates that functional self-regulation in the scope of SDT yields positive effects on the collective level. Mindfulness seems to give individuals a stronger orientation to their intrinsic values, which is positively related to more ecological and social behavior.

### Limitations and future research

We acknowledge certain limitations of the present study. First, it does not use longitudinal data to test the paths of the SEM. In cross-sectional studies, exposure and outcome are assessed at the same point in time, while in reality, any effect happens after its cause. In principle, longitudinal and experimental research designs are necessary to test for causalities empirically. But due to the complexity of the hypothesized causal model, it would be indeed difficult to test it with longitudinal data or in an experimental setting. Therefore, the approach was to firmly ground the hypothesized SEM with existing theoretical and empirical studies and test for its plausibility with cross-sectional data. In addition to the cross-sectional research design, a limitation of the study is that the sample used cannot be considered representative in any specific dimension and is limited by a self-selection bias of its participants. Furthermore, as only quantitative measurement instruments were used to measure the constructs, there could be a common method bias (Greve, 2006; Söhnchen, 2007). However, this study followed the four recommended methodological steps by Söhnchen (2007) when only singular data is used to prevent this bias.

## Conclusion

This study leveraged the knowledge base of self-determination theory to derive a causal model of healthy and effective self-regulation. The results support SDT's core propositions (Ryan et al., 2008; Schultz and Ryan, 2015) that mindfulness, intrinsic life-goals orientation, and autonomy of goals are essential for health, well-being, and effectiveness. Furthermore, a newly integrated construct, clarity about personal values, has been identified as essential. As the drafted model was only tested for falsification with cross-sectional data, we see it necessary to further validate the single parts of the model with longitudinal data and experimental settings. Our future work will contribute to this process by empirically developing and testing comprehensive interventions in the context of organizations that focus on mindfulness, intrinsic life-goals orientation, autonomous motivation, and clarity about personal values. Thus, we aim to put scientific knowledge into practice and further validate the discussed positive effects of specific self-regulatory processes on health, well-being, and effectiveness. As the discussed implications are theoretically and empirically well-grounded, we encourage testing and building on them in fields like psychotherapy, healthcare, organizations, sports, and education. We close with a quote by the psychologist Carl Gustav Jung, Adler, Jaffé and Hull (1973, p. 33) that represents the developed model well and that draws a vivid picture of the underlying philosophy:

“But your vision will become clear only when you can look into your own heart. ….*Who looks outside, dreams; who looks inside, awakes.”*

## Declarations

### Funding

Not applicable.

### Availability of data and material

The data can be made accessible by the authors if needed.

### Code availability

The calculations can be made accessible by the authors if needed.

### Ethics approval

Not applicable.

### Consent to participate

Not applicable.

### Consent for publication

Not applicable.

## Declaration of Competing Interest

Not applicable.
